# Development of an AlphaLISA assay for sensitive and accurate detection of influenza B virus

**DOI:** 10.3389/fmed.2023.1155551

**Published:** 2023-05-05

**Authors:** Huijun Zong, Shengwei Zhang, Xueyi Shang, Hua Jiang, Zhongpeng Zhao, Shaolong Chen, Xin Wang, Ye Wang, Yongqiang Jiang, Xinyu Li, Lingyun Tan, Peng Liu, Qingyu Lv, Yan Li

**Affiliations:** ^1^The PLA 307 Clinical College of Anhui Medical University, The Fifth Clinical Medical College of Anhui Medical University, Hefei, China; ^2^State Key Laboratory of Pathogen and Biosecurity, Academy of Military Medical Sciences, Beijing, China; ^3^Department of Intensive Care Unit, The Fifth Medical Center of Chinese PLA General Hospital, Beijing, China

**Keywords:** influenza B virus, AlphaLISA, LFIA, assay development, RT-qPCR

## Abstract

**Objective:**

Influenza B virus (IBV) is highly contagious, spreads rapidly, and causes seasonal epidemic respiratory disease in the human population, especially in immunocompromised people and young children. Clinical manifestations in this high-risk population are often more severe than in immunocompetent hosts and sometimes atypical. Therefore, rapid, and accurate detection of IBV is important.

**Methods:**

An amplified luminescent proximity homogeneous assay linked immunosorbent assay (AlphaLISA) was developed for detection of IBV by optimizing the ratio of IBV antibody-labeled receptor beads, streptavidin-conjugated donor beads and biotinylated IBV antibody, as well as the optimal temperature and time conditions for incubation. Assay sensitivity, specificity and reproducibility were evaluated. A total of 228 throat swab samples and inactivated influenza B virus were tested by AlphaLISA and lateral flow colloidal gold-based immunoassay (LFIA).

**Results:**

AlphaLISA produced the best results for detection of inactivated influenza B virus when IBV antibody-labeled acceptor beads were 50 μg/ mL, streptavidin-conjugated donor beads were 40 μg/mL, and biotinylated IBV antibody was 0.5 μg/mL at 37°C for 15–10 min. Under these conditions, AlphaLISA had a limit of detection of 0.24 ng/mL for the detection of influenza B nucleoprotein, did not cross react with other common respiratory viruses, and showed good reproducibility with inter-assay coefficient of variation (CV) and intra-assay CV < 5%. The results of 228 clinical throat swab samples showed good agreement between AlphaLISA and LFIA (Kappa = 0.982), and AlphaLISA showed better sensitivity than LFIA for detecting inactivated influenza B virus.

**Conclusion:**

AlphaLISA showed higher sensitivity and throughput in the detection of IBV and can be used for IBV diagnosis and epidemic control.

## 1. Introduction

Infections with Influenza A virus (IAV) and influenza B virus (IBV) are an important cause of respiratory tract disease in humans and cause yearly epidemics with significant morbidity and mortality. IAV infects a variety of hosts, including domestic and wild birds, humans and marine mammals and the antigen is readily mutated, predisposing to cause a pandemic. For IBV, it is primarily isolated from humans and in a rare event from a seal and is less antigenically mutated, which limits the risk of pandemic outbreaks (1, 2). In addition, studies on the epidemiology and clinical outcomes of IBV have been less thorough and IBV is frequently perceived as a less severe influenza infection (3). These factors make IBV less of a concern. It has been shown that IBV is more likely to spread in children (0–18 years) and if left untreated, can also trigger secondary bacterial infections, causing a range of complications (4). Data from latest years suggest that IBV infection in children accounts for 52% of all influenza related deaths (5). In addition, the symptoms caused by IBV are similar to those caused by IAV, including fever, headache, and muscle soreness. Therefore, it is difficult to distinguish IBV from other febrile illnesses on the basis of clinical presentation alone in younger children (6).

Early and appropriate antiviral therapy requires rapid, sensitive and specific diagnosis of IBV. Currently, several methods are commonly used for the detection of IBV, including viral culture, molecular diagnosis and immunological methods. Both viral culture and molecular diagnostic methods are considered the gold standard for diagnosing viral infections, but viral culture is time consuming and laborious, so it is not suitable for rapid screening of IBV. In molecular diagnostic methods, the development has been relatively mature, and methods have been developed to achieve rapid diagnosis of IBV. On the basis of PCR, the whole experimental time is significantly shortened. For example, CRISPR technology takes only 50–60 min (7) and the Alere i Influenza A & B system uses a technology for nucleic acid amplification at constant temperature that can detect influenza virus within 15 min (8).

Immunological methods are mainly based on serological antibody detection and rapid antigen detection. Serological detection includes hemagglutination inhibition test (9) and virus neutralization test (10), but these cannot detect early influenza B infection. Rapid antigen detection is the main method for rapid screening of IBV in clinical trials, and the lateral flow colloidal gold-based immunoassay (LFIA) is the most widely used. This method applies colloidal gold labeling and immunochromatography techniques with direct visualization of the results. When specific viral antigens are present in the specimen to be tested, antigen–antibody complexes are formed with colloidal gold-labeled antibodies in the front of the strip. The complex flows through the reagent membrane by chromatography, and is captured by the monoclonal antibody band, forming a double-antibody sandwich displayed as a violet-red band. The test can be completed within 15–20 min and has the advantages of convenient and rapid application (11). However, the main disadvantages are low sensitivity and vulnerability to environmental influences (12). Therefore, a homogeneous enzyme-linked immunoassay capable of rapid and sensitive detection of IBV was developed in the study.

Amplified luminescent proximity homogeneous assay linked immunosorbent assay (AlphaLISA) has always been an attractive method in the field of immunoassay due to its speed, simple operation and easy automation. AlphaLISA is a novel immunoassay technology, reducing hands-on and total assay time by separating bound and unbound assay components without multiple washes compared to previous enzyme linked immunosorbent assay (ELISA) and chemiluminescent immunoassays (CLIA). The method works by intermolecular interaction, that is, antigen–antibody specific binding makes the donor beads close to the acceptor beads (distance less than 200 nm). The donor beads are excited by a wavelength of 680 nm, and the photosensitive material in the donor beads converts oxygen in the surrounding environment to singlet oxygen, which diffuses to the acceptor beads. Following cascade reactions, the acceptor beads emit light at 615 nm (13). AlphaLISA developed based on the above principles has many advantages including less interference, high throughput, high sensitivity, and faster detection time (14). In this study, we developed an AlphaLISA assay for the detection of IBV and compared it with LFIA, which is widely used in clinical testing.

## 2. Materials and method

### 2.1. Samples, reagents and instruments

A total of 228 throat swab samples were collected from The Fifth Medical Center of Chinese PLA General Hospital from February 14 to July 11, 2019. Eu acceptor beads, streptavidin donor beads, and 1/2 AREAPLATE-96-well plates were purchased from PerkinElmer (United States). A pair of monoclonal antibodies (MAbs), 10-I55E and 10-I55B were purchased from Fitzgerald (United States). The inactivated influenza B virus was obtained from our laboratory and inactivated using β-propiolactone. The influenza B (B/Florida/4/2006) nucleoprotein (NP) with His tag was purchased from Sino Biological (Beijing, China). The influenza B antigen and the influenza A antigens (HIN1, H3N2, H5N1 and H7N9 subtypes) were purchased from Sinovac (Beijing, China). The antigens included respiratory syncytial virus (RSV), parainfluenza virus (PIV), and adenovirus (ADV) were purchased from Microbix (Toronto, Canada). Real time quantitative PCR (RT-qPCR) kits were purchased from BGI GBI (Beijing, China). TRNzol total RNA extraction kit was purchased from Tiangen (Beijing, China). LFIA detection kits were purchased from Abon Biopharm (Zhejiang, China). EZ-Link Sulfo-NHS-LC-Biotinylation kits and real-time PCR instrument were purchased from Thermo (United States). SPECTRAMAX i3 multiplate reader was purchased from Molecular Devices (United States). The study protocol has got approval of the ethics committee of the hospital (ky-2019-1-4). All methods were performed in accordance with the relevant guidelines and regulations.

### 2.2. AlphaLISA assay component preparation

The 500 μg receptor beads were washed by resuspension in PH7.4 phosphate buffered saline (PBS), the supernatant discarded, and 5 μL NaBH_3_CN (400 mM), 0.625 μL Tween-20 (10%), 50 μg IBV antibody and HEPES (pH7.4, 100 mM) were added in a total volume of 100 μL. 5 μL carboxymethoxyamine (65 mg/mL) was added after 24 h (h) rotating incubation at 37°C to block unreacted sites. After blocking for 1 h at 37°C, the beads were pelleted by centrifugation at 16,000 g for 15 min and the supernatant discarded. Beads were washed twice with Tris–HCl (pH 8.0, 100 mM), followed by resuspension in 100 μL PBS (pH 7.4, 10 mM) and stored at 4°C. Biotinylation of IBV antibody was performed according to the instructions of the biotin labeling kit.

### 2.3. Development of AlphaLISA assay

**Figure 1:**
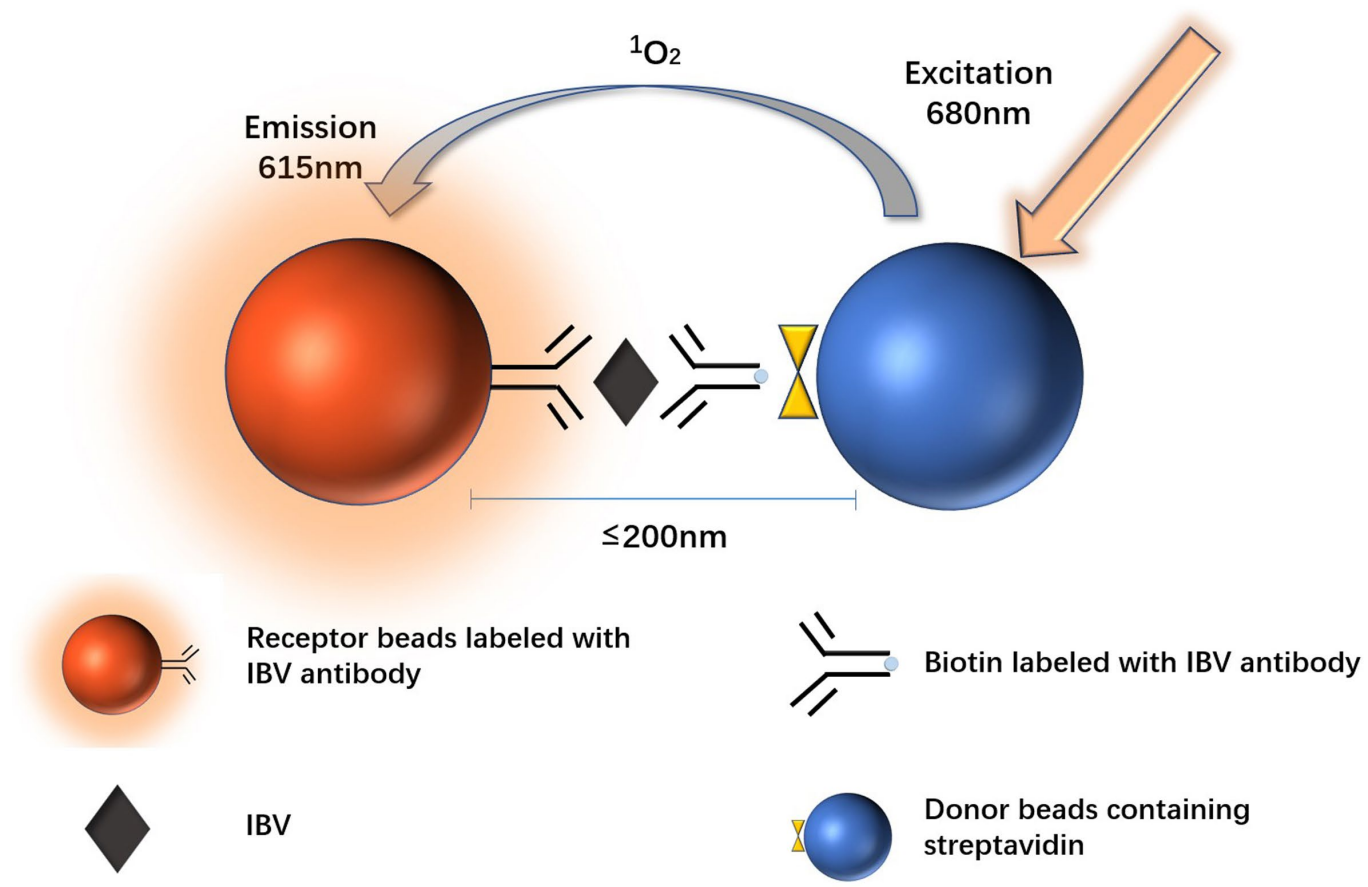
AlphaLISA assay principle.

The principle of AlphaLISA detection for IBV was based on a sandwich assay (Figure 1). A two-step assay procedure was used. 20 μL of a mixture solution of acceptor beads and biotinylated IBV antibody was added to 1/2 area – 96 well plates. And then 5 μL test sample (inactivated influenza B virus, influenza B NP, influenza B antigen or throat swab sample) was added to each well and incubated at 37°C for 15 min after shaking and mixing. Subsequently, 25 μL streptavidin-conjugated donor beads were added to the wells in the dark. After incubation for an additional 10 min at 37°C, the fluorescence signal was read by SPECTRAMAX i3 and the signal-to-noise ratio (the S/N ratio, the fluorescence signal of the test sample/the fluorescence signal of the blank control) was calculated.

Optimal concentrations of acceptor beads, donor beads, and biotinylated antibody were screened with the same incubation temperature (37°C) and time (15–10 min) conditions. The effects of: acceptor beads concentrations (100 μg/mL, 50 μg/mL, 25 μg/mL, 12.5 μg/mL or 6.25 μg/mL), biotinylated antibody dilutions (2 μg/mL, 1 μg/mL, 0.5 μg/mL, 0.25 μg/mL or 0.125 μg/mL) and donor beads at different concentrations (80 μg/mL, 40 μg/mL, 20 μg/mL, 10 μg/mL or 5 μg/mL) on the S/N ratio of 200-fold dilution of inactivated influenza B virus were compared. After optimization of beads and biotinylated antibody concentrations, the S/N ratios of 100-, 200-, 400-, and 800-fold dilutions of inactivated influenza B virus were tested at different incubation temperatures (37°C or 25°C) and different incubation time (the first incubation time-the second incubation time in minutes: 7.5–5, 15–10 or 30–20).

The influenza B NP was serially diluted 2-fold (0.06 ng/mL – 4,000 ng/mL) and then detected with AlphaLISA. Each concentration was repeated three times and the standard curve of influenza B antigen detection was modeled. The specificity of the AlphaLISA assay was evaluated and compared to other common respiratory viruses including IAV (HIN1, H3N2, H5N1 and H7N9 subtypes), RSV, PIV and ADV. The influenza B antigen and seven other respiratory viral antigens were diluted to 100 μg/mL, 10 μg/mL and 1 μg/mL in triplicate wells for each antigen dilution to compare the corresponding S/N ratio. 100- and 1,000-fold dilutions of inactivated influenza B virus was used to verify the repeatability of AlphaLISA. Three independent experiments in triplicate wells for each virus dilution were performed to validate inter-assay coefficient of variation (CV) of AlphaLISA; In one experiment, 12 replicate wells were tested for each virus dilution to verify the intra-assay CV.

### 2.4. LFIA experiment

The detection of LFIA was performed according to the kit instructions. 228 throat swab samples were tested in the hospital’s department of laboratory medicine. The detection of IBV was performed in our laboratory. Inactivated influenza B virus was diluted 1:100, 1:200, 1:400, 1:800, 1:1600, 1:3200, and 1:6400 using the lysate in the kit, and three replicates were made for each virus dilution gradient. The reagent strips were immersed in diluted virus diluent and the results were interpreted after 15 min incubation at room temperature.

### 2.5. RT-qPCR experiment

Fourteen throat swab samples were tested by RT-qPCR. RNA was extracted from samples using Trizol extraction. According to the RT-qPCR kit instructions, 19.7 μL of nucleic acid reaction solution, 0.3 μL of reverse transcriptase and 10 μL of extracted RNA were added to the reaction tube. The reaction tube was put into the fluorescence PCR detection instrument, and the program of fluorescence quantitative PCR instrument was set up according to the requirements: 50°C for 30 min 1 cycle; 95°C 15 min 1 cycle; 95°C 15 s, 58°C 45 s 40 cycle. The experimental process was carried out in accordance with the kit instructions and the instrument operation requirements.

### 2.6. Statistical analysis

Graphpad Prism 8.0, SPSS 26.0 and Sigmaplot 14.0 software were used for statistical analysis of all data. The student’s *t*-test was used for comparison between groups and the weighted kappa coefficient was used for concordance analysis. Diagnostic performance was evaluated using receiver operating characteristic (ROC) analysis, and the area under the curve (AUC) was calculated for comparison. When the Youden index reached the maximum value, the optimal cut-off value was determined. *p* < 0.05 was considered statistically significant.

## 3. Results

### 3.1. AlphaLISA optimization

**Figure 2:**
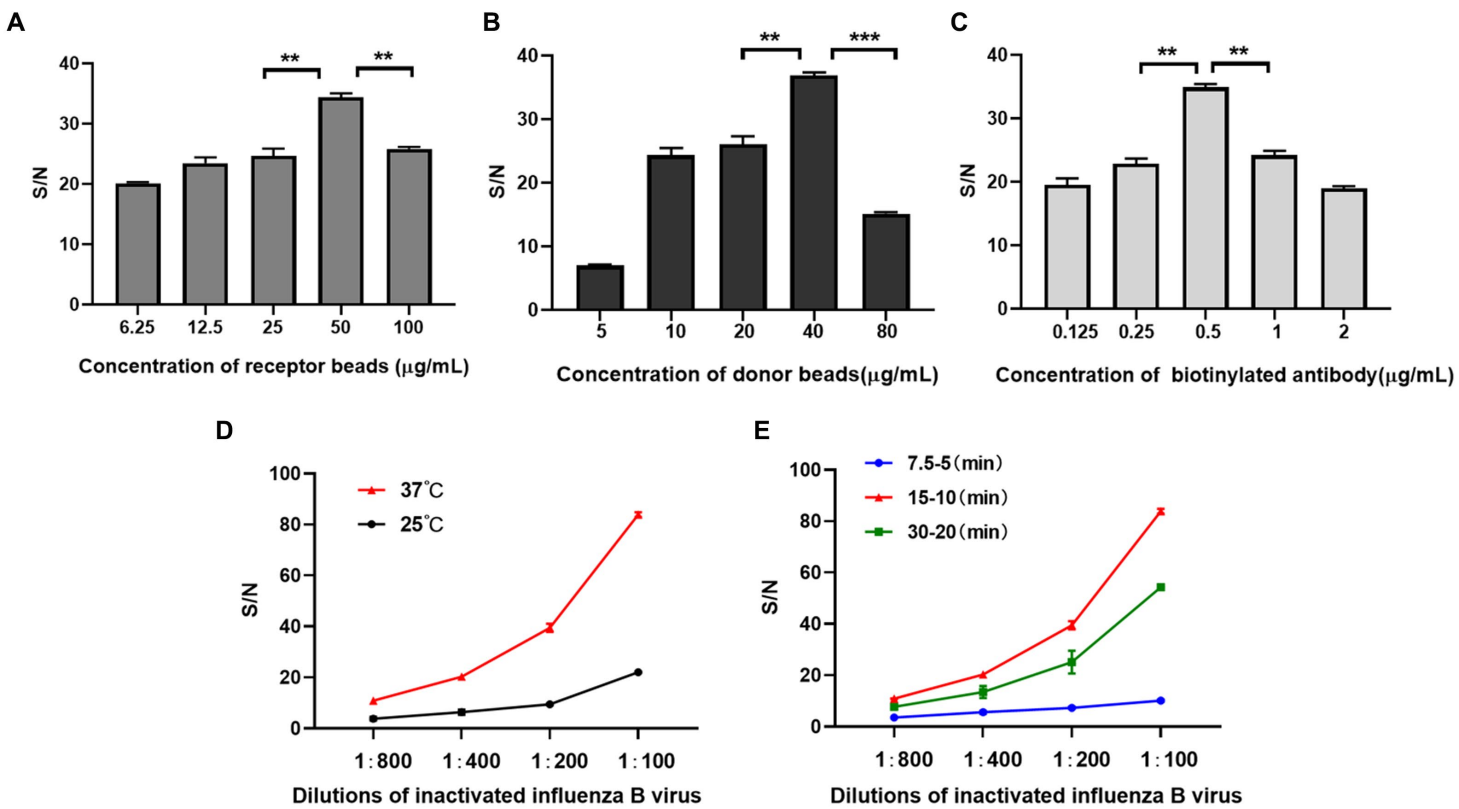
Development of AlphaLISA assay. **(A–C)** Optimization of acceptor beads concentration **(A)** donor beads concentration **(B)** and biotinylated IBV antibody **(C)**; **(D,E)** Optimization of incubation temperature**(D)** and time **(E)**. **(A–C)** is performed at 37°C and for 15–10 min. **(D,E)** were performed at 50 μg/mL for acceptor beads, 5 μg/mL for biotinylated antibody, and 40 μg/mL for donor beads. 7.5–5 (min), 15–10 (min) and 30–20 (min): the first incubation time-the second incubation time in minutes. **p < 0.01.

In order to avoid high background signal value caused by high concentration of reactant or weak signal value caused by low concentration of reactant, we tested the dilution concentrations of acceptor beads, biotinylated IBV antibody and donor beads. We found that the S/N ratio was maximum when acceptor beads were 50 μg/mL, biotinylated antibody was 0.5 μg/mL, and the donor beads were 40 μg/mL (Figures 2A–C).

In addition, for obtaining higher S/N ratio, we screened optimized incubation temperature and time for AlphaLISA. The temperature and time affect not only the binding of antibodies and analytes, but also the generation and diffusion of singlet oxygen (28). The results showed that when the incubation temperature was 37°C, the first incubation time was 15 min and the second incubation time was 10 min, the S/N ratios of all concentrations of inactivated influenza B virus were significantly higher than other temperatures and time (Figures 2D,E). So, this condition was selected for all subsequent experiments.

### 3.2. Sensitivity, specificity and repeatability

**Figure 3:**
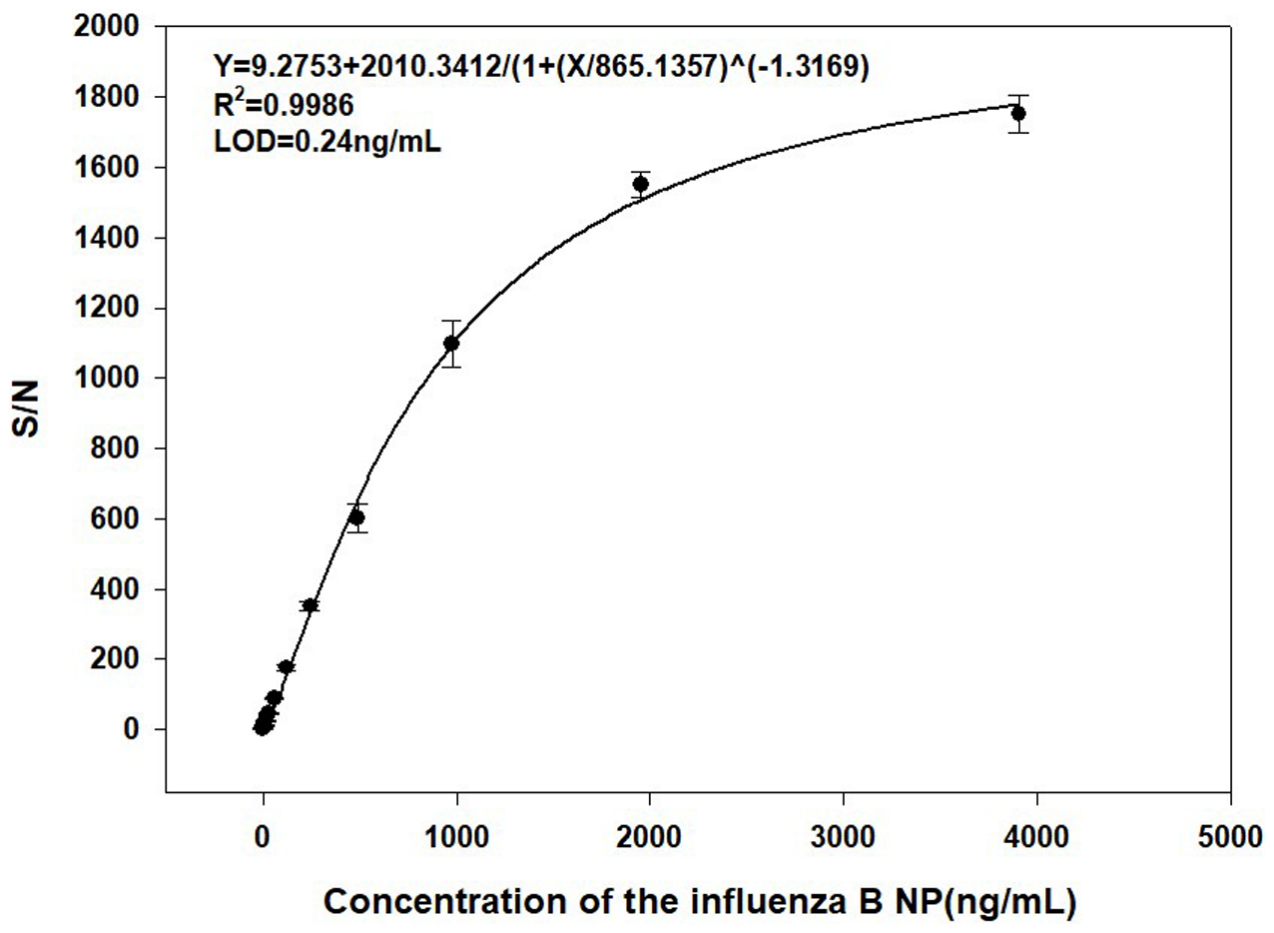
Standard curve for detection of the influenza B NP (2-fold serial dilution) by AlphaLISA (0.06 ng/mL – 4,000 ng/mL).

A standard curve was calculated with AlphaLISA signal (S/N ratio) as function of the influenza B NP (Figure 3). The results showed that the S/N ratio increased with influenza B NP concentration, and the four-parameter fitting curve was Y = 9.2753 + 2010.3412/[1 + (X/865.1357)] ^ (−1.3169) (*R*^2^ = 0.9986), with a limit of detection (LOD) of 0.24 ng/mL.

**Figure 4:**
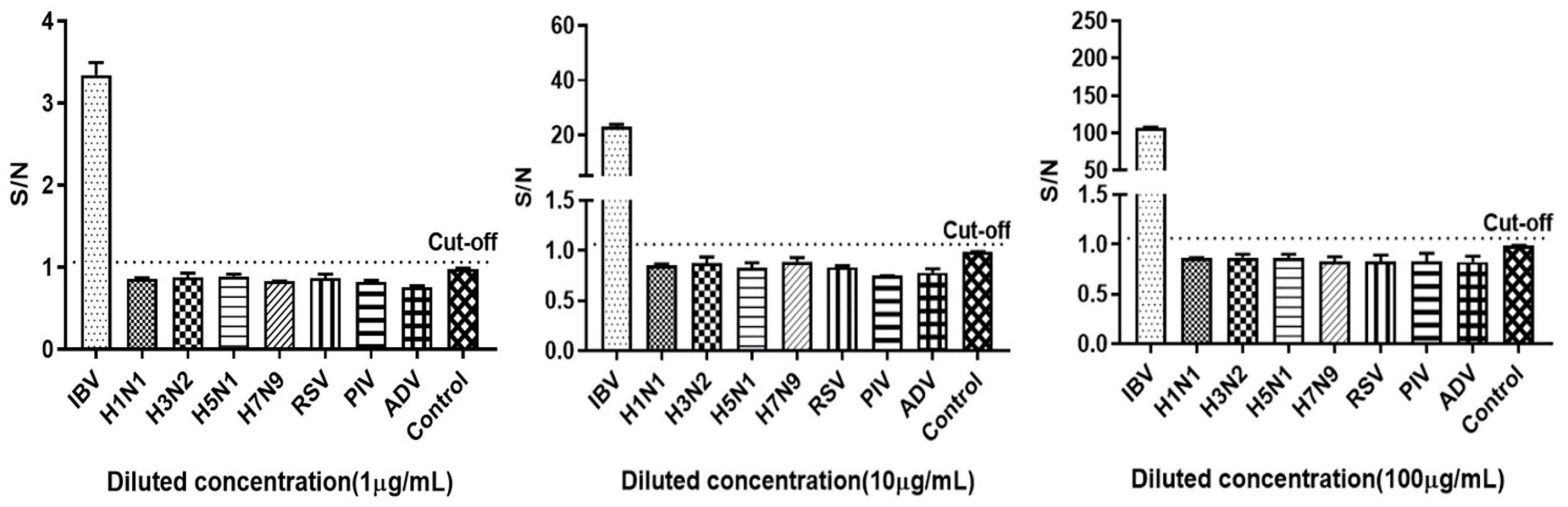
Specificity of the influenza B antigen detection using AlphaLISA. IBV: influenza B virus, HIN1, H3N2, H5N1 and H7N9: IAV subtypes, RSV: respiratory syncytial virus, PIV: parainfluenza virus, ADV: adenovirus. Control was AlphaLISA reaction buffer.

Since the clinical manifestations of IBV infection are nonspecifically different from those of other respiratory virus infections, and the therapeutic strategies corresponding to different viruses vary, the specificity of the method was validated. It showed that the S/N ratios for antigens of other seven respiratory viruses were all below the cut-off value, except for the influenza B antigen (Figure 4, the cut-off value was 1.06, derived from subsequent ROC curve). This suggested that the method was specific and did not cross-react with other respiratory viruses.

The results of repeatability experiments showed that the inter-assay CV and intra-assay CV were all under 5%, suggesting good repeatability of AlphaLISA (Table 1), probably driven the methods technical ease and lack of any washing steps, which greatly reduced interference caused by human manipulation.

**Table 1:** Inter-assay and intra-assay precision.

virus dilution gradients	Inter-assay variation (%)	Intra-assay variation (%)
10^−2^	2.86	2.40
10^−3^	3.48	2.71

### 3.3. Comparison of the AlphaLISA and LFIA

**Figure 5:**
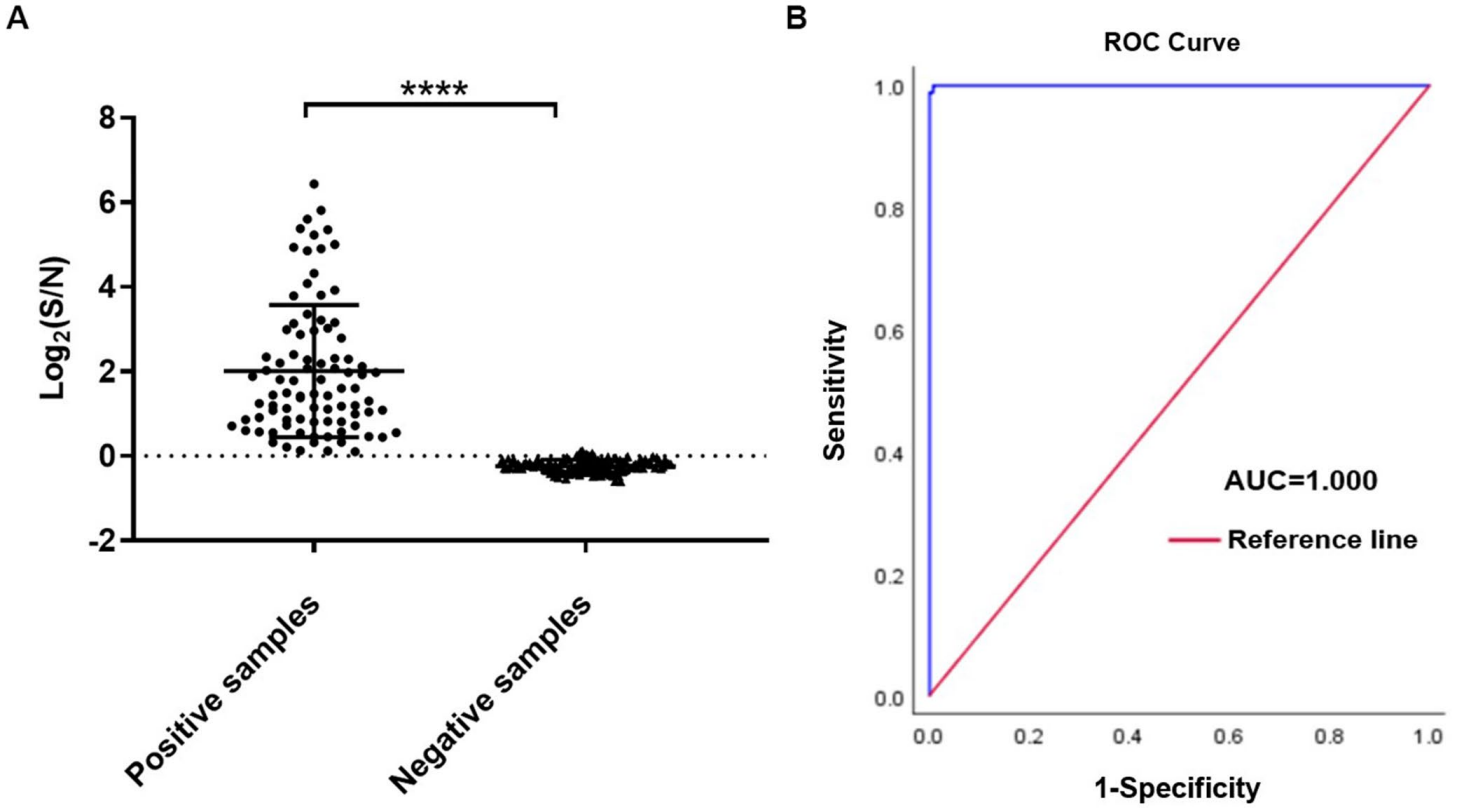
AlphaLISA results for throat swab samples. **(A)** Sample test results for positive versus negative; **(B)** ROC curve for LFIA vs. AlphaLISA (*n* = 228). ****p < 0.0001.

There were 89 positive samples and 139 negative samples for IBV in 228 clinical throat swab samples tested by LFIA. Meanwhile we tested the above 228 throat swab samples with AlphaLISA, and the S/N ratios were calculated. Mean ± SEM (standard error of the mean) of positive and negative samples were 13.91 ± 0.16 and 0.77 ± 0.09, respectively. The results showed that there was a significant statistical difference in S/N ratio between positive and negative samples (*p* < 0.0001) (Figure 5A). The ROC curve was plotted using LFIA as the reference method and showed high agreement between AlphaLISA and LFIA with an area under the curve AUC of 1 (Figure 5B). According to the ROC curve, the cut-off value was determined to be 1.06. When the S/N ratio of the test sample was above 1.06, it was considered positive, and below 1.06 was considered negative. The comparative results of AlphaLISA and LFIA for detecting throat swab samples are shown in Table 2. The overall agreement, positive agreement, and negative agreement of AlphaLISA compared with LFIA were 99.12, 100.00, and 98.56%, respectively. The weighted kappa coefficient was 0.982 (*p* < 0.001), and the asymptotic 95% confidence interval was 0.956–1.007. Two samples showed negative results using LFIA but positive results using AlphaLISA. Given that RT-qPCR is the gold standard for the detection of IBV and offers high sensitivity and good specificity, we performed RT-qPCR validation on these two samples, both of which showed positive by RT-qPCR, validating the AlphaLISA results. To further validate the accuracy of AlphaLISA, 12 additional throat swab samples with the S/N ratios close to cut-off value were tested by RT-qPCR, which again were concordant with a positive RT-qPCR test (Table 3).

**Table 2:** Comparison of the AlphaLISA and LFIA results.

LFIA	AlphaLISA			Concordance rate
	Positive	Negative	Total	
Positive	89	0	89	100.00%
Negative	2	137	139	98.56%
Total	91	118	228	99.12%

**Table 3:** RT-qPCR results of 14 throat swab samples with the S/N ratios close to cut-off value by AlpahLISA.

LFIA	AlphaLISA		RT-qPCR	
	S/N ratio	Result	CT value	Result
P	1.48	P	37.00	P
P	1.46	P	34.85	P
P	1.37	P	31.04	P
P	1.36	P	34.61	P
P	1.25	P	34.08	P
P	1.16	P	35.64	P
P	1.07	P	33.37	P
N*	1.08	P	36.71	P
N*	1.07	P	36.24	P
N	1.03	N	–	N
N	0.92	N	–	N
N	0.88	N	–	N
N	0.82	N	–	N
N	0.73	N	–	N

P: positive, N: negative. *The sample showed negative results using LFIA but positive results using AlphaLISA.

**Figure 6:**
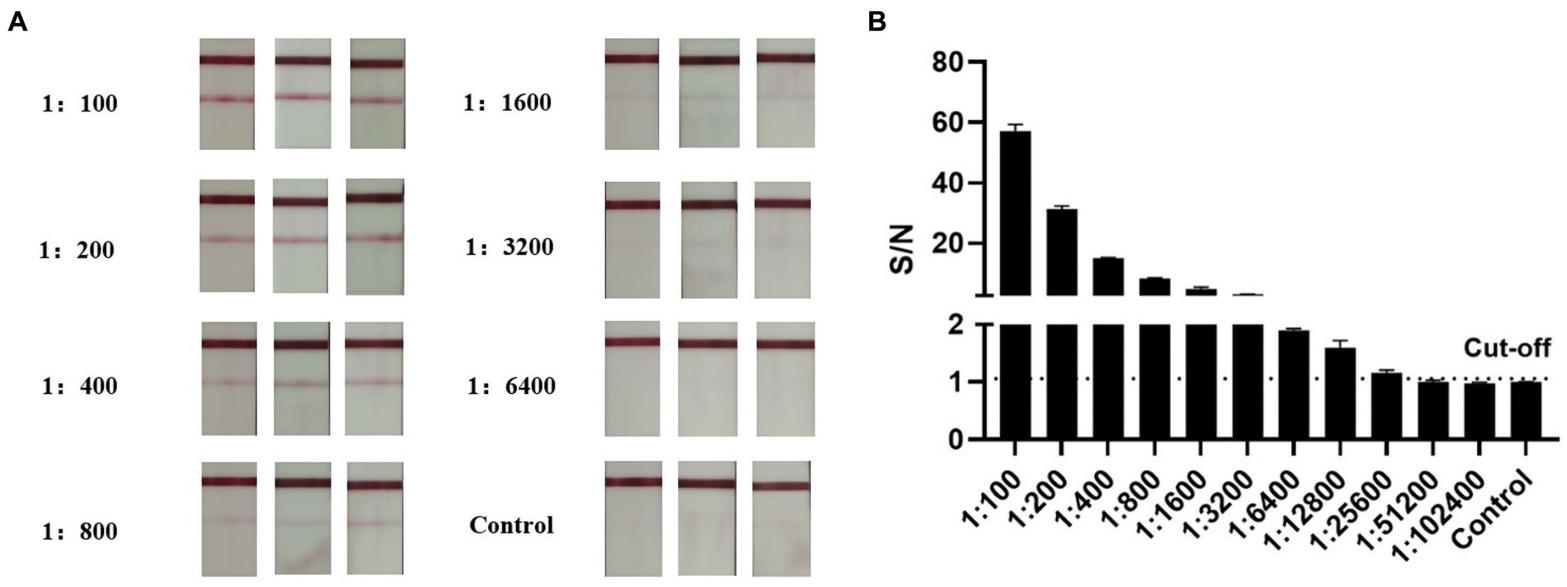
Comparison of AlphaLISA and LFIA sensitivity. **(A)** LFIA for detection of inactivated influenza B virus at different dilutions (2-fold serial dilution, 1:100–1:6,400). Tests were performed in triplicates for each dilution, and the control was colloidal gold lysate. **(B)** AlphaLISA detection of inactivated influenza B virus at different dilutions (2-fold serial dilution, 1:100–1:102,400). Tests were performed in triplicates for each dilution, and the control was AlphaLISA reaction buffer.

To further compare the sensitivity of the AlphaLISA method with LFIA, inactivated influenza B virus was detected at different dilution gradients. We found that AlphaLISA could detect diluted inactivated influenza B virus at a dilution of 1:25,600, whereas LFIA could only detect inactivated influenza B virus in 1:3,200 dilution (Figure 6). AlphaLISA had higher sensitivity and was able to detect lower IBV titers than LFIA. This likely explains the two positive throat swab samples detected by AlphaLISA but negative by LFIA.

## 4. Discussion

IBV accounts for a significant proportion of seasonal influenza, especially in susceptible groups such as children, adolescents and the elderly. Because IBV is restricted to humans and there are no persistent animal hosts, there are currently very limited data on which adaptive features IBV has acquired to achieve continuous human-to-human transmission. One of the major proteins known to be involved in host adaptation to IBV is the hemagglutinin (HA), which enables virus attachment to host cells and uptake *via* endocytosis playing a critical role during viral infection (15). IBV HA is clearly adaptable to the mildly acidic pH and low temperatures of the human upper airways, these distinct properties likely reflect host adaptation resulting from sustained presence of this respiratory pathogen in the human population (16). Furthermore, HA protein genetic information is prone to change and evolutionary drift which may lead to periodic epidemics (17). During viral epidemics, rapid initiation of antiviral therapy and prophylaxis to effectively control infection requires rapid and accurate viral detection. Susceptibility to IBV infection varies in the population. In immunocompromised people, even low viral loads may cause mild to severe symptoms. In immunocompetent people, IBV has a 1 to 3 days incubation period, marked by often neglected mild symptoms. Additionally, some patients may have a low viral load due to viral shedding when they present with clinical symptoms (within 12 h after onset or fever) (18). Therefore, a highly sensitive assays with good specificity is more conducive for the detection of low titer IBV, ensuring the appropriate use of anti-influenza drugs in people with severe symptoms and effective epidemic control in general population.

AlphaLISA is a homogeneous light-stimulated chemiluminescence sandwich immunoassay. At present, this assay is useful for detection of viral and food toxins as well as human molecular proteins. It showed good specificity and sensitivity in the detection of SARS-CoV-2 and African swine fever virus (19, 20).In the detection of staphylococcal enterotoxin, T2 toxin, and Shiga toxin 2 in food products, and it has been shown to have good accuracy with low sample sizes and no interference by contaminants (21–23). Furthermore, it has a wide linear range as showed by the detection of adrenocorticotropic hormone (24). Therefore, we applied AlphaLISA to the rapid detection of IBV.

The ideal approach for pathogen detection should be rapid, sensitive, specific, affordable, sometimes instrument-free and suitable for Point-of-care-testing (POCT) (25). Currently, the modalities used to achieve faster and more precise control of IBV are mainly through LFIA screening and RT-qPCR diagnosis. LFIA, as an immunoassay technique, has the advantages of convenient use, low cost and intuitive detection results. However, this method still has the problem of low sensitivity. At the same time, the result is mainly judged by visual observation, which will be influenced by certain subjective judgment. In addition, LFIA is mainly used for qualitative detection and rarely for quantitative detection (26). RT-qPCR, as the gold standard for the detection of IBV, has a very high sensitivity. However, this method is cumbersome and generally requires centralized diagnosis laboratories and skilled operators (27). AlphaLISA developed in this study belongs to one of the immunoassay techniques. Compared with LFIA, AlphaLISA showed superior sensitivity, which is mainly due to 60,000 singlet oxygen generated by irradiated donor beads, inducing significant signal amplification after interaction with acceptor beads (28). In addition, high antibody density on beads could further improve sensitivity if needed (29). And this method determines negative and positive depending on the number of fluorescence values, the results are more objective, and could be used for quantitation of analytes. AlphaLISA still requires the instrument to read the results compared to LFIA. However, this method performs following simple ‘mix-and-measure’ protocols and is ideal suited for miniaturization and automation (30). Therefore, this assay has potential on-site rapid detection capabilities. AlphaLISA, although less sensitive than RT-qPCR, has the advantage of simplicity, rapidity, and does not require professional operators and a strictly clean experimental environment. IBV is prevalent almost every year in areas with high population density and scarcity of medical resources (31). In areas where RT-qPCR testing is not available, AlphaLISA has the advantage of high sensitivity and throughput over LFIA, enabling large area screening of IBV positive patients at different stages of infection. It facilitates epidemic control in social groups and early diagnosis and treatment of individuals.

## 5. Conclusion

In summary, AlphaLISA had good sensitivity, specificity and repeatability in the detection of IBV and had the advantages of easy operation and rapid reaction.

## Data availability statement

The raw data supporting the conclusions of this article will be made available by the authors, without undue reservation.

## Ethics statement

The studies involving human participants were reviewed and approved by The Fifth Medical Center of Chinese PLA General Hospital. Written informed consent for participation was not required for this study in accordance with the national legislation and the institutional requirements.

## Author contributions

HZ, SZ, XS, HJ, ZZ, SC, XW, YW, YJ, XL, LT, PL, QL, and YL participated in the study concept and design. HZ, PL, and QL performed the data analysis and wrote the draft. SZ performed the sample collection. All authors contributed to the article and approved the submitted version.

## Funding

This work was funded by the National Science and Technology Major Projects (2018ZX10711001003 and 2018ZX10713002002004).

## Conflict of interest

The authors declare that the research was conducted in the absence of any commercial or financial relationships that could be construed as a potential conflict of interest.

## Publisher’s note

All claims expressed in this article are solely those of the authors and do not necessarily represent those of their affiliated organizations, or those of the publisher, the editors and the reviewers. Any product that may be evaluated in this article, or claim that may be made by its manufacturer, is not guaranteed or endorsed by the publisher.
